# 3D laparoscopy does not reduce operative duration or errors in day-case laparoscopic cholecystectomy: a randomised controlled trial

**DOI:** 10.1007/s00464-019-06961-1

**Published:** 2019-07-16

**Authors:** Katie E. Schwab, Nathan J. Curtis, Martin B. Whyte, Ralph V. Smith, Timothy A. Rockall, Karen Ballard, Iain C. Jourdan

**Affiliations:** 1 Minimal Access Therapy Training Unit, University of Surrey, Daphne Jackson Road, Guildford, UK; 2 Department of General Surgery, Royal Surrey County Hospital, Egerton Road, Guildford, UK; 3 Department of Surgery, Royal Bournemouth and Christchurch Hospitals NHS Foundation Trust, Castle Lane East, Bournemouth, UK; 4 Department of Surgery and Cancer, Imperial College London, Praed Street, London, UK; 5 Department of General Surgery, Yeovil District Hospital NHS Foundation Trust, Higher Kingston, Yeovil, UK; 6 University of Surrey, Guildford, UK; 7 Department of Surgery, Frimley Park Hospital, Portsmouth Rd, Frimley, UK

**Keywords:** 3D, Three-dimensional, Laparoscopic, Cholecystectomy, Gallbladder, Trial

## Abstract

**Background:**

Contemporary 3D platforms have overcome past deficiencies. Available trainee and laboratory studies suggest stereoscopic imaging improves performance but there is little clinical data or studies assessing specialists. We aimed to determine whether stereoscopic (3D) laparoscopic systems reduce operative time and number of intraoperative errors during specialist-performed laparoscopic cholecystectomy (LC).

**Methods:**

A parallel arm (1:1) randomised controlled trial comparing 2D and 3D passive-polarised laparoscopic systems in day-case LC using was performed. Eleven consultant surgeons that had each performed > 200 LC (including > 10 3D LC) participated. Cases were video recorded and a four-point difficulty grade applied. The primary outcome was overall operative time. Subtask time and the number of intraoperative consequential errors as identified by two blinded assessors using a hierarchical task analysis and the observational clinical human reliability analysis technique formed secondary endpoints.

**Results:**

112 patients were randomised. There was no difference in operative time between 2D and 3D LC (23:14 min (± 10:52) vs. 20:17 (± 9:10), absolute difference − 14.6%, *p* = 0.148) although 3D surgery was significantly quicker in difficulty grade 3 and 4 cases (30:23 min (± 9:24), vs. 18:02 (± 7:56), *p* < 0.001). No differences in overall error count was seen (total 47, median 1, range 0–4 vs. 45, 1, 0–3, *p* = 0.62) although there were significantly fewer 3D gallbladder perforations (15 vs. 6, *p* = 0.034).

**Conclusion:**

3D laparoscopy did not reduce overall operative time or error frequency in laparoscopic cholecystectomies performed by specialist surgeons. 3D reduced Calot’s dissection time and operative time in complex cases as well as the incidence of iatrogenic gallbladder perforation (NCT01930344).

**Electronic supplementary material:**

The online version of this article (10.1007/s00464-019-06961-1) contains supplementary material, which is available to authorized users.

The short-term patient benefits provided by laparoscopy present surgical challenges. The inherent loss of depth perception has been a source of focus for industry and led to the development and marketing of three-dimensional (3D) systems. Adoption of early 3D platforms was limited by poor image resolution and user side effects [1]. Technological advancements in processing and visual presentation have revived surgical interest as contemporary systems appear to have addressed these issues without increasing cognitive load [2–4].

Systematic reviews have concluded that 3D systems appear to improve task and operative time and reduce performance errors when compared to 2D laparoscopy [5, 6]. However, the role of 3D laparoscopy in routine clinical practice has not been sufficiently evaluated as the available literature predominantly focusses on trainee performance of box trainer tasks or comparative studies with significant methodological concerns reported [2, 5, 7]. Ex-vivo specialist performance has also been seen to improve but the paucity of clinical studies mean it is unclear if this translates to the delivery of laparoscopic interventions in the operating room [8, 9].

Therefore, we aimed to investigate whether the reported benefits in surgical efficiency and error reduction from the use of 3D laparoscopic systems were present in routine clinical practice. We hypothesised that 3D laparoscopy reduces the operative time and number of intraoperative errors enacted during laparoscopic cholecystectomy (LC) compared to the current 2D reference standard.

## Methods

A single centre, parallel arm (1:1) randomised controlled trial (RCT) was designed in keeping with an IDEAL stage IIb exploration study as well as recommendations for 3D laparoscopic studies and the CONSORT principles (Supplementary Table 1) [5, 7, 10]. Research ethical approval was granted by the UK National Health Service East Midlands committee (ref: 13/EM/0092) and local research department (ref: 13SURN0004). This trial is registered (NCT01930344).

### Patient eligibility criteria

Inclusion criteria were patients listed for elective day-case LC, age 18–80 and provision of written informed consent. All patients received an abdominal ultrasound scan and liver function tests. Additional pre-operative imaging was performed at the discretion of the responsible surgeon. Exclusion criteria were previous upper abdominal surgery, known common bile duct stones, cholecystectomy planned with any other combined surgical procedure (including bile duct exploration), planned overnight or post-operative inpatient stay and inability or refusal to provide written informed consent. In keeping with standard practice for day-case surgery, patients with an American Society of Anaesthesiologists physical classification score > 2, age > 80 or a body mass index ≥ 35 kg/m^2^ were also excluded.

### Surgeon eligibility criteria and stereopsis testing

All consultant surgeons performing laparoscopic cholecystectomy at the Royal Surrey County Hospital UK were approached to participate. All had performed a minimum of 200 independent day-case elective LC including at least 10 3D laparoscopic cases. Surgeons received a written information pack and face-to-face trial briefing. To enrol, surgeons had to provide written informed consent and undergo stereopsis testing (Wirt Fly sterotest (Stereo Optical Inc^®^, Chicago, IL, USA). This evaluates both gross stereopsis (2500 to 1200 s of arc) and fine depth perception (800 to 40 s). All participants were seen to have normal stereo acuity (defined as ≤ 120 s of arc).

### Equipment, set-up and procedures

All cases were performed using either a Karl Storz IMAGE1 S D3-Link™ system with zero-degree 10 mm TIPCAM^®^1 SPIES 3D video laparoscopes or Olympus 3DV-190 system with Endoeye™ 10 mm rigid 3D laparoscopes. Images were displayed on liquid crystal display high definition screens (Panasonic EJ-MDA32E-K, Panasonic^®^ Europe, Wiesbaden, Germany or Sony LMD-2451MT, Sony Europe Ltd., Surrey, UK) and viewed with passive polarising glasses. 2D cases used identical equipment. Screen positioning and viewing distance was at the discretion of each surgical team. All surgeons used a four-port technique with dissection performed with Maryland forceps or hook diathermy. To maximise recruitment, generalisability of results and ethical and surgeon acceptability, no constraint on anaesthetic techniques, timing of surgery, precise operative technique, instrument use or intraoperative decision were made. On table cholangiography was performed at the discretion of the operating surgeon. All perioperative care proceeded as per local site policies.

### Randomisation procedure

Upon provision of consent, each patient was allocated a unique trial ID as sole identifier. This was matched to a block plan of two options randomised over 120 stems which had been generated pre-trial. These results were placed in sealed envelopes, labelled by study ID, in the theatre admissions unit and opened during induction of anaesthesia maintaining allocation concealment. Given the sample size, further stratification was not undertaken.

### Observational clinical human reliability analysis (OCHRA)

To assess whether 3D imaging influences intraoperative performance, analysis of intervention delivery was performed. The OCHRA technique assesses the interaction of humans with complex systems with the aim of highlighting the mode and mechanism behind error occurrence and increase awareness and data to aid future avoidance [11–14]. OCHRA involves structured analysis of operative case video to identify errors. In this study, consequential errors were defined “adverse events that required extra unplanned step(s) to correct or manage, or deviation from the standard operative task”. OCHRA has been successfully applied to a variety of laparoscopic procedures including LC, cases performed within RCTs, as well as assessment of specialist surgical performance with reliability and face, construct and concurrent validity established [11–13, 15–18].

As surgical interventions can be considered as a series of interconnecting steps which can be further broken down into sub-tasks, a previously reported LC hierarchical task analysis (HTA) was utilised [14]. Each step was analysed for consequential errors and a model of error production applied to each event. Additionally, the nature, timepoint and brief description of each error event was captured and categorised using a pre-defined list of external error modes (EEM, Table 3) [14, 19]. OCHRA analysis was performed independently by two reviewers after completion of human factors and OCHRA training from expert assessors. Reviewers were blinded to trial arm, surgeon, date of surgery and all patient and clinical details.

### Endpoints and sample size

The primary end-point was total surgical time (from grasping and elevation of the gallbladder fundus until complete detachment of the gallbladder from the liver bed) (Table 2). As there was no prior 3D LC research to guide sample size calculations, mean operative times from a meta-analysis of 12 four-port LC RCTs (all in 2D) were reviewed showing a weighted mean time of 45.8 min [20]. A minimally clinically important difference of 12 min (25%) was adopted. Using a two-sided test (*α* 0.05, *β* 0.8), a minimum of 50 patients per arm would be required. Allowing for attrition, a recruitment target of 120 was selected. Pre-defined secondary endpoints were time for each operative task zone (Table 2) and number of consequential errors enacted.

### Data collection

The integrated stack systems (AIDA™), Karl Storz Endoskopy, Germany and image management hub IMH-20, Olympus Europe, Hamburg, Germany) were used to record unedited, deidentified procedures. Irrespective of trial arm, all videos were recorded in 2D without sound or extra corporeal views. To reduce heterogeneity and ensure comparable assessment, video files were edited using iMovie for MacOS (v10, Apple Inc™, Cupertino, CA, USA) to show the three task sections forming the primary endpoint which were left unaltered (Table 2). If an on table cholangiogram was performed, this segment was removed from the video and not analysed nor contributed to any endpoint. Edited videos were collated and issued for assessment at least 3 months after surgery.

### Case difficulty grading

Procedural difficulty varies and can influence surgical time and error rate [21]. Therefore, an intraoperative assessment of macroscopic gallbladder pathology was made using a validated scale: grade 1 (thin-walled gallbladder, no adhesions), grade 2 (filmy gallbladder adhesions), grade 3 (thick-walled or surrounded by adhesions) or grade 4 (dense adhesions, attachment of adjacent organs or gallbladder mucocele or empyema) [22].

### Statistical analyses

The data were analysed using SPSS (v25.0; SPSS Inc, Chicago, IL, USA). Data were tested for normality with the Shapiro–Wilk test and detrended Q–Q plots and compared with parametric or non-parametric tests as appropriate. Unpaired *T* Test, Mann–Whitney *U* and Kruskal–Wallis testing were used to compare means/medians from normal and non-normally distributed populations respectively. For categorical data, association between groups was analysed with cross-tabulation, Fisher’s exact test or Pearson’s Chi squared. Operative time was log transformed and 2D and 3D cases compared with ANCOVA using gallbladder case grade as a covariant. The degree of inter-rater agreement between OCHRA observers was compared using Cohen’s kappa (*κ*) with the 95% confidence interval (CI) calculated using the standard error of κ. Data are displayed as means with standard deviations unless otherwise specified. Comparative results are reported as (2D vs. 3D) throughout. Intention to treat data is presented although where a complete case recording was necessary for analysis, a per protocol approach was used. Statistical significance was defined as *p* < 0.05.

## Results

**Fig. 1:**
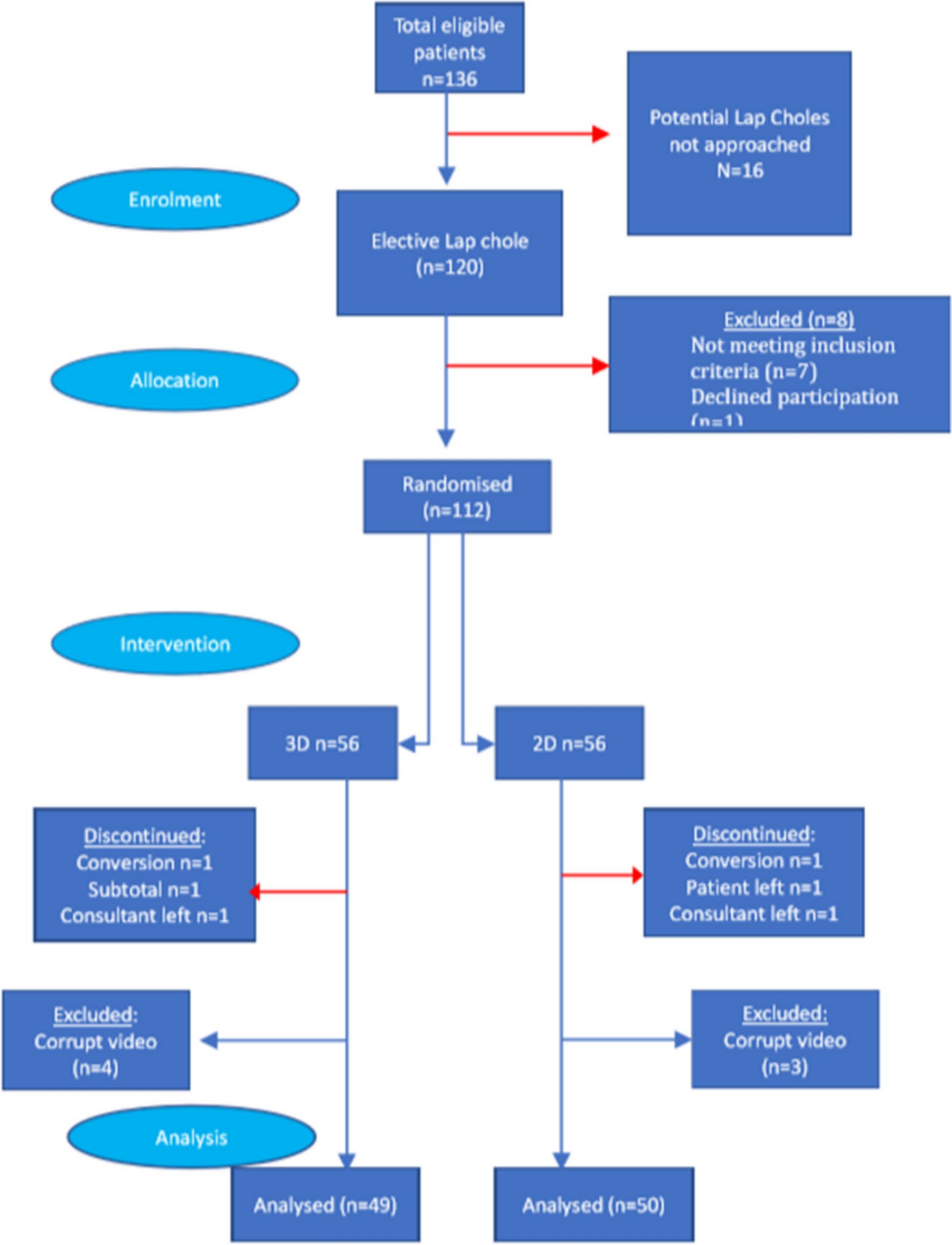
Trial CONSORT diagram. 136 day-case LC were performed during the study period. 16 (11.7%) were not approached due to researcher unavailability. Of the 120 patients screened for eligibility, 113 approached with 112 consenting to trial entry (99.1%, 82.4% of all unit LC). Attrition was 11%, equal between the arms and inside study design. Incomplete video was the main reason

136 patients underwent day-case LC between May 2013 and September 2014. Trial CONSORT diagram is shown in Fig. 1. Of the 120 patients screened, 113 were eligible and 112 (99%) recruited. The attrition rate was 11.6% and equal between arms (incomplete or corrupt case video (*n* = 7), conversion to open surgery (*n* = 2, 1.8%, 1 per arm), LC performed by a trainee (*n* = 2, one per arm), subtotal cholecystectomy (*n* = 1) and one patient declined surgery). In total, 99 patients underwent day-case LC with a complete video available.

74% of participants were female. Mean age was 52 years (range 24–80). Baseline patient characteristics were evenly distributed between the trial arms (Table 1). Biliary colic was the most common indication for surgery (52%) followed by past cholecystitis (39%), gallstone pancreatitis (5%), gallbladder polyp (2%) and passed common bile duct stone (1%).

**Table 1:** Patient demographics, indication and LC case difficulties

	2D			3D			*p*
	Mean (SD)	Count	Column *N* (%)	Mean (SD)	Count	Column *N* (%)	
Age	53 (14)			51 (16)			0.679
Gallbladder case difficulty grade							
1		17	34		23	46.9	0.991
2		22	44		7	14.3	
3		7	14		14	28.6	
4		4	8		5	10.2	
Sex							
Females		34	68		40	81.6	0.119
Males		16	32		9	18.4	
Indication for surgery							
Biliary colic		26	52		26	54.2	0.397
Cholecystitis		18	36		20	41.7	
Gallstone pancreatitis		2	4		2	4.2	
Gallbladder polyp		3	6		0		
Passed common bile duct stone		1	2		0		

As might be expected from the inclusion criteria there were significantly more lower grade cases (*p* < 0.001)

### Operative case complexity

In keeping with study inclusion criteria overall there were significantly higher numbers of low-grade cases (grade 1 *n* = 40, grade 2 *n* = 29, grade 3 *n* = 21 and grade 4 *n* = 9; *p* < 0.001, Table 1). Overall gallbladder grades were equal between the trial arms (*p* = 0.991) although the 3D arm contained a significantly higher proportion of grade 3 and 4 cases (*p* = 0.011). No surgeon reported any eye symptoms or complaints during the duration of this study.

### Operative time

**Fig. 2:**
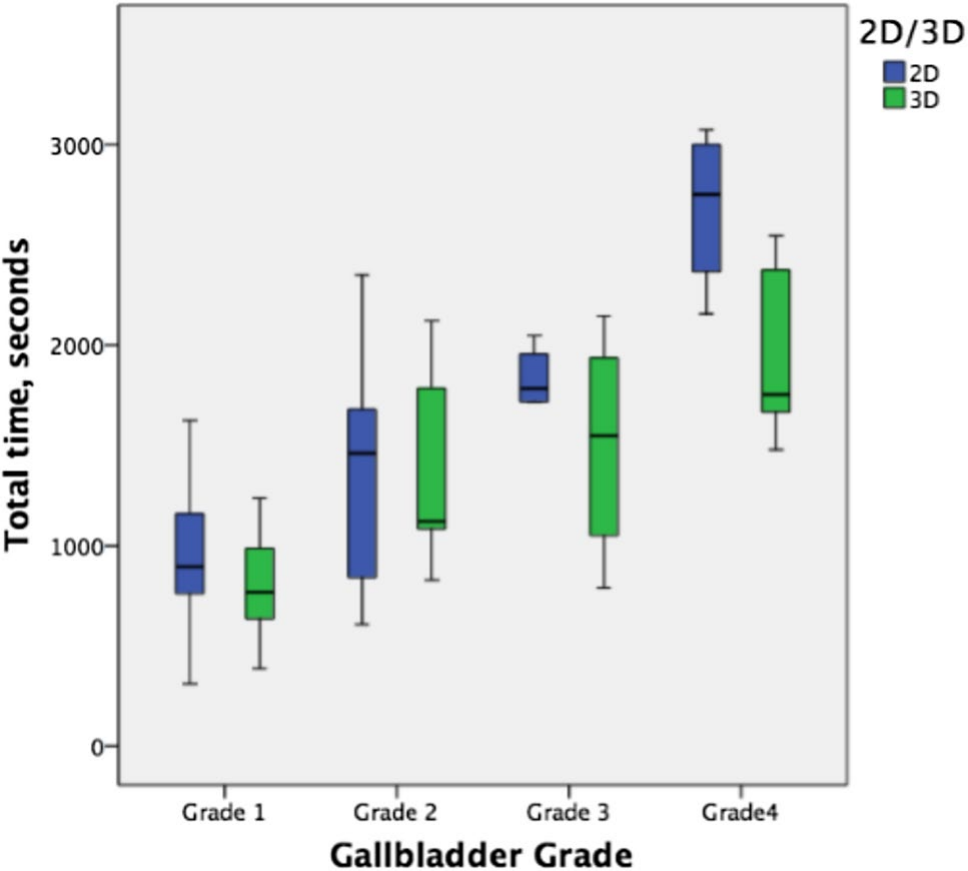
Gallbladder grade is seen to have a larger impact on operative time. 3D was significantly faster for grade 3 and 4 cases

The mean time to complete a 3D LC was 20:17 (± 9:10) minutes compared to 23:14 (± 10:52), absolute difference − 14.6%, *p* = 0.148. Operative time increased with each gallbladder grade (grade 1 = 14:24 min (± 4:42) vs. grade 2 = 23:01 (± 8:50) vs. grade 3 = 27:07 (± 7:28) vs. grade 4 = 38:02 (± 9.22), *p* < 0.001, Fig. 2). Grade 3 and 4 cases were performed significantly faster in 3D (18:02 (± 9:24) vs. 30:23, *p* < 0.001).

Following natural log transformation of the three hierarchical task zones, a normal distribution was seen (Shapiro–Wilk test *p* = 0.23). ANCOVA analysis showed that higher gallbladder grades were significantly associated with longer operative times (partial *η*^2^ = 0.507, *p* < 0.001, Table 2). Overall operative times were reduced with 3D systems, but this did not reach statistical significance with a small effect size observed (partial *η*^2^ = 0.039, *p* = 0.056). HTA defined operative task 1–3 breakdown showed a statistically significant time reduction for task one when 3D was used (median 724 s (interquartile range 380–1068) vs. 540 (288–792, *p* = 0.013)) but not task 2 or 3 (Table 2).

**Table 2:** Hierarchical task analysis used in the trial. Adapted from the reports by Joice et al. [14] and Tang et al. [11]

Task	Start point	End point	Trial arm	Median (IQR) (s)	*p*	Dependent variable of natural log of time. Partial *η*^2^	*p*
1. Dissection of Calot’s triangle	Grasping of fundus and elevating	Clear identification of cystic artery and duct	2D	724 (380–1068)	0.061	0.063	**0.013**
			3D	540 (288–792)			
2. Clipping and dividing cystic artery and cystic duct	Appearance of clip applicator	Cystic artery and duct divided	2D	140.5 (44–237)	0.23	0.007	0.414
			3D	182 (76–288)			
3. Detaching gallbladder from liver bed	At completion of cystic artery and duct division	Gallbladder fully removed from liver bed	2D	324.5 (203.5–445.5)	0.894	0.000	0.941
			3D	281 (196–366)			
Total operative time	Grasping of fundus and elevating	Gallbladder fully removed from liver bed	2D	1287 (834–1783)	0.148	0.039	0.056
			3D	1070 (790–1616)			

Time (s) is displayed. No differences are seen in the direct comparison but after natural transformation a significant difference in Calot’s dissection alone is seen

### OCHRA error analysis

99 complete videos were available for analysis comprising 2156 min of LC surgery. A total of 92 intraoperative errors were identified (median 1 per case, range 0–4). No differences were seen between the 2D and 3D arms (median 1, range 0–4, total 47, cases with error(s) 66%, vs. 1, 0–3, 45, 61%, *p* = 0.62). Excellent inter-rater reliability was seen (κ = 0.81 (95% CI 0.7–0.92), *p* < 0.001, Supplementary Table 1).

Errors were more frequent in higher grade gallbladder cases (grade 1 = 0.75 per case, grade 2 = 0.97, grade 3 = 0.86 and grade 4 = 1.78; *p* < 0.001) but when controlling for grade, no differences were seen between 2D and 3D LC (*p* = 0.879). There was no difference in error counts between the three surgical task phases (39 vs. 25 vs. 27, *p* = 0.181). OCHRA categorical data is displayed in Table 3. Executional errors (EEM 7–10) accounted for 75% of error events with no difference between the trial arms (*p* = 0.186). With regards to consequential errors, gallbladder perforations were significantly reduced in the 3D cases (15 vs. 6, *p* = 0.034) otherwise no differences were seen in the external error modes or specific nature and frequency of errors between the trial arms (Table 3).

**Table 3:** External error modes—this model considers errors as being either ‘inter-step’ (the correct steps being performed in the correct order; error modes 1–6), or ‘intra-step’ (the execution, or lack of, the subtask; error modes 7–10). Adapted from Cuschieri et al. [32]

External error mode	2D error count	3D error count	Error event enacted	Inter-step errors (EEM 1–6)		Intra-step errors (EEM 7–10)	
				2D	3D	2D	3D
Step is not done	1	1	Calot’s triangle bleeding	1	1		
Step is partially completed	5	7	Bleeding from gallbladder	0	0		
Step is repeated	2	4	Injury to liver	0	0		
Second step is done in addition	1	2	Clip application error	8	13		
Second step is done instead of first step	0	0	Gall bladder perforation	0	0		
Step is done out of sequence	0	0					
Step is done with too much force/speed/depth/distance/time/rotation	20	17	Calot’s triangle bleeding			8	6
Step is done with too little force/speed/depth/distance/time/rotation	3	3	Bleeding from gallbladder			8	12
Step is done in wrong orientation/direction/point in space	15	11	Injury to liver			5	5
Step is done on/with the wrong object	0	0	Clip application error			2	2
			Gall bladder perforation			**15**	**6**
Sum	47	45		9	14	38	31

OCHRA results are shown. No difference in error modes are seen between the 2D and 3D cases. Intra-step (executional, EEM 7–10) errors accounted for 75% of error events. The only significant difference observed was fewer gallbladder perforations with 3D surgery (*p* = 0.034)

## Discussion

The minimal access surgery revolution is dependent on advancements of technology and technique. Stereoptic laparoscopy overcomes the inherent loss of depth perception associated with 2D systems. Laboratory-based studies with trainee and specialist participants have suggested 3D allows faster performance with fewer errors, but their findings cannot be assumed to be applicable to actual operating theatre performance [3]. As there is increasing uptake of 3D systems without supportive evidence it could be argued that the innovation has outpaced surgical evaluation.

We performed an RCT assessing the impact on time and errors using LC as a representative high-volume index procedure and additionally as the Cochrane 2D/3D LC review was based on a single 1998 RCT [23, 24]. We incorporated all methodological recommendations for 3D studies advocated by the available 3D systematic reviews and evaluation of surgery as defined by the IDEAL collaborative [5, 7, 10].

We saw a non-clinically nor statistically significant 3-min reduction in operative time with 3D arm primarily from faster Calot’s triangle dissection. Case difficulty could represent a major confounding variable within LC studies. We applied a four-point difficulty classification based on direct intraoperative visualisation recently validation in our population and saw a significant increase in operation time with each additional grade [22]. Although there were only 21 grade 3 and 9 grade 4 cases in the trial and there was a higher proportion of these within the 3D arm, 3D was significantly faster in these potentially challenging cases with a 40% operative time reduction.

Operative duration alone is an insufficient measure of surgical performance. Direct observation of the intraoperative period was performed independently by two blinded reviewers using the structured, validated OCHRA technique as this is where any impact of imaging technology is most likely to be evident. Provision of stereoscopic imaging did not alter the number of enacted error events or the underlying external error mode. Significantly fewer perforations of the gallbladder, which can present significant consequences to patients [25], occurred in 3D cases as the only difference between the arms.

Our findings, which could be expected to be widely applicable, are comparable with the original 1998 and very recent 2D/3D LC RCT reports although our notable absence of subjectively reported side effects and eye symptoms suggests contemporary 3D technology holds improved usability [24, 26]. Our data suggests specialist LC performance was not altered by the technology used, possibly as the participating surgeon experience allowed them to overcome the loss of depth perception. There is supportive evidence for 3D laparoscopy for trainees, but the specialist surgeon proficiency gain curve has not been defined [3].

Surgical technologies undergo intensive development and safety testing but efficacy assessment including within-operating room settings are not mandatory for licencing which contrasts with regulatory requirements in other healthcare areas. Surgical intervention studies can present challenges not encountered in other areas of clinical research [10]. Our study is strengthened by the randomised design that aimed to reduce potential bias that can influence comparative studies of surgical technology. Robotics represents another area of contemporary debate where high level evidence has contradicted previous favourable reports with concerns raised regarding the influence of industry funding on study reporting [27, 28]. A number of questions on 3D laparoscopy remain, particularly regarding health economic data. Although not formally studied here, our results suggest no meaningful difference in resources would be expected.

We successfully recruited to time and target and observed very high recruitment of eligible patients, however, our findings should be considered in light of some limitations. As there were no contemporary 3D LC data available at the time of study design we based our power calculation on external 2D LC RCT reports with differing operative time definitions [20]. Our operative duration was considerably faster in both 2D and 3D LC which likely invalidates this calculation and risks introduction of type II errors although the small observed time difference is unlikely to be clinically significant or relevant to healthcare providers. Ideally, trial equipment would have been standardised but regular simultaneous day-case LC theatre lists necessitated use of a second 3D comparable passive polarising technology platform although this was from a second manufacturer. Reassuringly no outcome differences were seen between the two systems.

We observed that case difficulty was a major cofounding variable with a larger impact on operative time than the imaging system used. The case complexity distributions were unequal between the trial arms with more complex cases in the 3D arm which affected the primary endpoint comparison. As there is no reliable method to pre-operatively predict case difficulty, we would advise future researchers to consider on table randomisation which allows stratification for the observed difficulty. This has been successfully used in surgical interventional RCTs to ensure participant and procedure eligibility [29]. Although we complied with the CONSORT criteria and 80% of elective UK LC are now performed as day-case procedures [30], selection bias cannot be fully excluded as more comorbid patients, potentially difficult LC cases (including the acute cases and the emergent setting) were excluded. Finally, case video review does not capture human factors that may influence surgical performance and procedure duration [31].

## Conclusion

3D laparoscopy did not reduce overall operative time or error frequency in laparoscopic cholecystectomies performed by specialist surgeons. 3D reduced Calot’s dissection time and operative time in complex cases as well as the incidence of iatrogenic gallbladder perforation (NCT01930344).

## Electronic supplementary material

Below is the link to the electronic supplementary material.

Supplementary Table 1 OCHRA inter-rater reliability. Cohen Kappa calculation table to compare agreement and non-agreement between the two observers. Excellent agreement is observed ĸ = 0.81 (95%CI 0.7-0.92) (DOCX 11 kb)
