# Supplementary material for: 3D laparoscopy does not reduce operative duration or errors in day-case laparoscopic cholecystectomy: a randomised controlled trial
Source: Surg Endosc. 2019 Jul 16;34(4):1745–53. doi: 10.1007/s00464-019-06961-1 (PMC7093411; doi:10.1007/s00464-019-06961-1)
Supplement: Supplementary file 1 — OCHRA inter-rater reliability. Cohen Kappa calculation table to compare agreement and non-agreement between the two observers. Excellent agreement is observed ĸ = 0.81 (95%CI 0.7-0.92) (DOCX 11 kb) [file 464_2019_6961_MOESM1_ESM.docx]

|  | | Observer 1 | | |
| --- | --- | --- | --- | --- |
|  |  | Error | No error | Total |
| Observer 2 | Error | 84 | 4 | 88 |
|  | No error | 7 | 35 | 42 |
|  | Total | 91 | 39 | 130 |

Pr(a) = 84+35/130 = 0.92

Pr(e) = (88/130 x91/130) + (42/130x39/130) = 0.58

Cohen’s Kappa = (0.92–0.58) / (1-0.58) = **0.81** (95% Confidence Interval 0.70-0.92).

Supplementary table 1
